# The Landscape of IFN/ISG Signaling in HIV-1-Infected Macrophages and Its Possible Role in the HIV-1 Latency

**DOI:** 10.3390/cells10092378

**Published:** 2021-09-09

**Authors:** Masyelly Rojas, Patricia Luz-Crawford, Ricardo Soto-Rifo, Sebastián Reyes-Cerpa, Daniela Toro-Ascuy

**Affiliations:** 1 Facultad de Ciencias de la Salud, Instituto de Ciencias Biomédicas, Universidad Autónoma de Chile, Santiago 8910060, Chile; masyellyrojas@gmail.com; 2 Centro de Investigación e Innovación Biomédica, Facultad de Medicina, Universidad de los Andes, Santiago 7620001, Chile; patricia.luzc@gmail.com; 3 Molecular and Cellular Virology Laboratory, Virology Program, Faculty of Medicine, Institute of Biomedical Sciences, Universidad of Chile, Santiago 8389100, Chile; rsotorifo@med.uchile.cl; 4 Centro de Genómica y Bioinformática, Facultad de Ciencias, Universidad Mayor, Santiago 8580745, Chile; 5 Escuela de Biotecnología, Facultad de Ciencias, Universidad Mayor, Santiago 8580745, Chile

**Keywords:** HIV, latent HIV-1 reservoir, macrophages, IFN/ISG response, epitranscriptomic regulation

## Abstract

A key characteristic of Human immunodeficiency virus type 1 (HIV-1) infection is the generation of latent viral reservoirs, which have been associated with chronic immune activation and sustained inflammation. Macrophages play a protagonist role in this context since they are persistently infected while being a major effector of the innate immune response through the generation of type-I interferons (type I IFN) and IFN-stimulated genes (ISGs). The balance in the IFN signaling and the ISG induction is critical to promote a successful HIV-1 infection. Classically, the IFNs response is fine-tuned by opposing promotive and suppressive signals. In this context, it was described that HIV-1-infected macrophages can also synthesize some antiviral effector ISGs and, positive and negative regulators of the IFN/ISG signaling. Recently, epitranscriptomic regulatory mechanisms were described, being the N6-methylation (m6A) modification on mRNAs one of the most relevant. The epitranscriptomic regulation can affect not only IFN/ISG signaling, but also type I IFN expression, and viral fitness through modifications to HIV-1 RNA. Thus, the establishment of replication-competent latent HIV-1 infected macrophages may be due to non-classical mechanisms of type I IFN that modulate the activation of the IFN/ISG signaling network.

## 1. Current Status

Human immunodeficiency virus (HIV) is the etiologic agent of acquired human immunodeficiency syndrome (AIDS) and represents one of the most extensively studied pathogens. Since its identification in the early 1980s, HIV/AIDS has reached pandemic levels, with more than 38 million people reported to be living with the virus worldwide contributing to the deaths of hundreds of thousands of people annually [1,2].

HIV infection is now considered a chronic disease and not a lethal one, and approximately 60% of HIV-positive individuals receive antiretroviral therapy (ART) [2,3]. Although ART can suppress viral replication, it does not eliminate the cells harboring replication-competent latent virus [4,5]. The suspension of ART may result in viral rebound, even after years of treatment [6]. In patients undergoing ART and who have undetectable levels of circulating virus, HIV-type 1 (HIV-1) continues to traffic between tissue compartments, and a combination of dynamic and spatial processes allows the virus to persist within the infected host [7].

HIV-1 reservoirs are defined as all the infected cells and tissues containing any form of HIV-1 persistence that can contribute to its pathogenicity [8,9]. Their existence is determined by the permissiveness of a cell to HIV-1 infection and its ability to become latent, cells that possess viral DNA integrated into transcriptionally active sites on host chromosomes and harbor proviral DNA that upon reactivation generates replication component virus [9]. At the same time, HIV-1 tropism is governed by the distribution of cellular proteins that the virus requires to enter the cell, including CD4, the receptor for HIV-1, and its chemokine co-receptors CCR5, CXCR4, and CCR3. The distribution of these receptors allows HIV-1 to infect not only CD4^+^ T cells, but also cells from the myeloid lineage such as macrophages, dendritic cells, and microglia [10,11,12,13], any of which can eventually become HIV-1 reservoirs.

The seeding of the HIV-1 reservoir happens within the first 3 to 5 days of infection [4]. Once established, the reservoirs persist in different compartments of the body, although primarily in the intestine and central nervous system (CNS) [4]. Virus entry into the CNS occurs 4 to 8 days after peripheral infection, likely through infected monocytes and lymphocytes, and is established in macrophages and microglia where it remains replication-competent despite long periods of suppression resulting from ART [14,15,16]. HIV-1 entry into the CNS leads to neurological abnormalities, termed HIV-associated neurocognitive disorders (HAND), which manifests despite successful ART in 30% of cases [17]. Viral proteins such as Tat and gp120, together with the expression of cytokines/chemokines and the activation of adhesion molecules, drive the additional recruitment of monocytes and lymphocytes, leading to chronic neuroinflammation that is conducive to the production of neurotoxic factors and, eventually, neuronal dysfunction and cell death [4,18,19,20].

HIV-1 persistence favors chronic immune activation and sustained inflammation, both of which are hallmarks of HIV-1 infection and the main cause of HIV-associated non-AIDS complications (HANA) [3,21,22]. Indeed, in HIV-positive individuals, aberrant immune activation with persistent inflammation is associated with an excess risk of morbidity and mortality [23,24,25]. Moreover, it is well-documented that the generation of an inflammatory environment stimulates viral dissemination and the formation of latent viral reservoirs [23]. Acute infection (in the absence of ART) results in a cytokine storm, which is associated with the production of IFN-α and IL-15, followed by IL-10, TNF-α, IP-10, IFN-γ, and IL-6 [26]. Similarly, during the chronic inflammatory state (in absence of ART), there is also an increase in the serum levels of cytokines, chemokines, pro-inflammatory soluble mediators, acute phase proteins, microbial sensors, and coagulation factors [23]. Although the source of these cytokines is not clear, the local production of these mediators in multiple sites probably contributes to maintaining their high levels. Moreover, it has even been suggested that there is an inflammatory set point in an untreated infection that persists after ART-mediated virologic suppression [27]. Accordingly, in CD4^+^ HIV-1-infected cells, the transcription factor hypoxia-inducible factor 1 (HIF-1) promotes viral replication and inflammation by promoting the release of extracellular vesicles. In turn, this triggers the secretion of inflammatory mediators, resulting in the secretion of IFN-γ by bystander CD4^+^ T cells and IL-1 and IL-6 by bystander macrophages, thus contributing to HIV-1 pathogenesis [21].

## 2. Macrophages as HIV-1 Reservoirs

Myeloid cells have the potential to become a latent reservoir of HIV-1 and produce infectious virus [28]. However, the mechanisms involved in the control of latency in myeloid cells remain unclear and are likely distinct from those in CD4^+^ T cells [29,30]. Monocytes have an average lifetime of one day in the circulation and cannot be considered long-term HIV-1 reservoirs [31,32]. Nevertheless, the different monocyte subpopulations are phenotypically and functionally heterogeneous with regard to immune function and can differentiate to macrophages or dendritic cells in inflamed tissues [8]. In this context, several studies have identified proviral DNA in macrophages from the lung [33], duodenum [34], gut-associated lymphoid tissue (GALT) [35], astrocytes, and microglia [36]. However, the presence of proviral DNA does not necessarily indicate direct infection as this genetic material can result from the phagocytosis of debris from CD4^+^ T cells [37]. Moreover, engulfment was shown to lead to productive macrophage infection in vitro [38].

Until recently, it was assumed that HIV-1-infected macrophages did not show apparent signs of cytotoxicity but sustained a steady production of HIV-1 without undergoing cell death [39]. However, more recent research has clarified that while acute HIV-1 infection of human microglia/macrophages results in massive apoptosis in vitro, a small population of HIV-1-infected cells survive the infection, silence viral replication, and can reactivate viral production following specific treatments, such as N-hydroxy-N′- phenyl-octanediamide, suberoylanilide hydroxamic acid (SAHA), Phytohemagglutinin (PHA), methamphetamine, lipopolysaccharide (LPS) and the combination of TNF-α and IFN-γ [40]. The newly recognized longevity and self-renewing potential of CNS-associated macrophages [41] suggests that they may be a reservoir for viral infection and renders them a preferred site for replication, in agreement with previous reports [42,43]. Investigating the role of tissue-resident macrophages in HIV-1 infection is challenging. Since, to study the interaction between the virus and macrophages resident in specific tissues, it has become necessary to use primary cultures derived from tissues such as fetal brains or post-mortem analysis of patients with AIDS [44]. Nevertheless, the results of studies in simian immunodeficiency viruses (SIV) involving macaque [15] and humanized mouse model [39] have predicted that microglia and perivascular macrophages are likely to be infected by HIV-1, which could contribute to HIV-1 persistence [15,39] independently of T cells [45]. A delayed viral rebound was observed in 33% of humanized myeloid-only mice seven weeks after ART interruption, which is consistent with the establishment of persistent infection in macrophages [46], and with the participation of macrophages as HIV-1 reservoir. Using highly sensitive in situ hybridization techniques, Ko et al. recently demonstrated that, in patients undergoing ART and in whom the viral load was effectively suppressed, HIV-1 DNA can be identified in macrophages/microglia of the CNS but not in astrocytes. The authors were also able to detect viral RNA, indicative of low-level viral reactivation and/or focal replication [47]. Combined, these results suggested that brain macrophages are an important reservoir of HIV-1. Nevertheless, the molecular mechanism underlying requires further investigation.

It remains unknown if macrophages can support latency similar to that seen for memory CD4^+^ T cells [9]. Castellano et al. established an in vitro primary culture model to determine the mechanism by which immune cells resist HIV-1-induced apoptosis, thereby becoming reservoirs [40]. They concluded that latently HIV-1-infected macrophages/microglia, which act as HIV-1 reservoirs, block a very early step of apoptosis that involves the accumulation of Bim protein (a pro-apoptotic negative regulator of Bcl-2) in the mitochondria [40]. Additionally, these cells display altered metabolism and are characterized by mitochondrial fusion, lipid accumulation, and reduced mitochondrial ATP production, resulting in a unique metabolic signature [48].

Alvarez-Carbonell and colleagues established an immortalized human microglial cell line that allowed to generate stable cell lines latently infected with HIV-1 proviruses [49], representing an important tool to study latent reservoirs and microglial cell function during HIV-1 infection [50].

## 3. Intracellular Innate Immune Response in HIV-1-Infected Macrophages

During the infection cycle, HIV-1 must overcome several cytoplasmic barriers associated with the innate immune response [51], even in cells permissive to infection (e.g., CD4^+^ T cells and macrophages) [52,53]. Prominent among these barriers is IFN-mediated immunity, which provides a robust first line of antiviral defense [54,55,56].

### 3.1. The IFN/ISG Signaling Network and HIV-1

The IFNs comprise a family of pro-inflammatory, immunomodulatory, pleiotropic cytokines that induce an antiviral state through the upregulation of hundreds of IFN-stimulated genes (ISGs) [57]. IFNs are classifieds into three different groups—type I (α, β, δ, κ, ε, τ, ω, and z), type II (γ), and type III (λ1, λ2, and λ3)—according to the structure of their receptors, among other factors [51]. All nucleated cells can synthesize type I IFN when pathogen-associated molecular patterns (PAMPs) are detected through germline-encoded pattern recognition receptors (PRRs) [58]. In the case of the response against HIV-1, PRR activation leads to the activation of innate immune-associated signaling pathways and the consequent expression of a plethora of proteins that can restrict almost all states of the HIV-1 replication cycle [51,58,59].

The main intracellular receptors of the innate immune response include three types: Toll-like receptors (TLRs), retinoic acid-inducible gene 1 (RIG-1)-like receptors (RLRs), and nucleotide oligomerization domain (NOD)-like receptors (NLR) [60,61]. Recently, Meier et al. have suggested that HIV-1 encodes multiple uridine-rich oligonucleotides that can be TLR7/8 ligands and induce a strong MyD88-dependent plasmacytoid dendritic cell and monocyte activation, as well as accessory cell-dependent T-cell activation and thereby contribute to the immune activation observed during viremic HIV-1 infection [62]. Similarly, sensing of HIV-1 RNA and induction of a type I IFN response can be triggered by RIG-1, which is able to recognize dimeric or monomeric forms of HIV-1 RNA, as well as secondary structured HIV-1-derived RNA in infected macrophages [63,64].

Once all three IFN types of production are induced by pathogen detection, IFN molecules bind to their receptors on the cell surface and signal in a paracrine/autocrine fashion through the Janus kinase/signal transducer and activator of transcription (JAK/STAT) pathway [56,65]. This leads to the formation of a transcription complex that recognizes IFN-stimulated response elements (ISREs) located in ISG promoters and activates their transcription [56,66]. To date, more than one thousand ISGs have been identified [53] and can be classified into the following three groups based on their function:

- Antiviral effectors: There is a plethora of HIV-1-induced antiviral effectors. For example, some molecules with anti-HIV-1 activity, such as the apolipoprotein B mRNA editing complex 3 (APOBEC3) family of proteins, act by binding to HIV-1 RNA and inducing hypermutation in the newly synthesized HIV-1 DNA early in the viral cycle, which restricts provirus replication [51,67]. Members of the tripartite motif (TRIM) family of proteins, such as TRIM5α, enhance the fragmentation of viral cores, preventing HIV-1 cDNA synthesis [57,68]. Sterile alpha motif and histidine–aspartate domain-containing protein 1 (SAMHD1) can restrict viral replication by reducing the number of nucleotides available for viral DNA synthesis [69,70]. Some members of the dynamin GTPase superfamily, such as myxovirus resistance 2 (Mx2), prevent the nuclear import and integration of viral DNA [57,71] while tetherin inhibits the release of the virus [51,72].
- Positive regulators of IFN signaling: These include molecules such as IFN regulatory factor 3 (IRF3) [73], 1, 2, and 7 [74]; cyclic GMP-AMP synthase (cGAS) [75]; melanoma differentiation-associated gene 5 (MDA5) [76]; and RIG-1 [77]. These proteins act as sensors, second messengers, or effector molecules and contribute to the antiviral response. Some lentiviruses, including HIV-1, can induce the production of several positive regulators of IFN signaling, such as IRF1, IRF2, IRF7, cGAS, MDA5, RIG-1, and IFN-γ-inducible protein 16 (IFI16), which confer protection against infection in a species- and cell-type-dependent manner [78].
- Negative regulators of IFN signaling: These include suppressor of cytokine signaling (SOCS) proteins, which inhibit JAK/STAT signaling [79], or ubiquitin-specific peptidase 18 (USP18) [80], which induces a state of desensitization in the target cell, thereby rendering the cell refractory to IFN stimulation [56]. HIV-1 infection can reportedly induce SOCS1, which, in turn, can affect the innate and adaptive immunity responses [81]. Another study revealed that, in CD4^+^ T cells of HIV-infected patients, SOCS1/3 mRNA levels were upregulated, whereas their protein levels were downregulated, which may explain the lack of attenuation of the JAK/STAT pathway [82]. Similarly, it was proposed that the reduced viability of memory CD4^+^ T cells induced by type I IFN during HIV-infection is USP18/protein kinase B (AKT)/phosphatase and tensin homolog (PTEN)-dependent [83]. In macrophages and dendritic cells, USP18 can promote HIV-1 replication by enhancing reverse transcription through the downregulation of the expression of p21 (a cyclin-dependent kinase inhibitor), which correlates with the antiviral-inactive form of SAMHD1 [84].

The antiviral immune response is highly efficient and relies on the function of ISGs that employ multiple pathways and a complex network of interactions with different cellular proteins that contribute to its function [85]. Hubel et al. investigated the protein–protein interaction network (interactome) of ISGs and identified regulators of viral immunity and processes related to the immune system [85]. In this article, the authors report the interaction between ISGs and various cellular proteins, which are described with a role in signaling induced by HIV-1 or even with previous reported interaction with the viral proteins, stands out bone marrow stromal antigen 2 (BST2) [86], Programmed cell death 6 (PDCD6) [87], and lectin galactoside-binding soluble 3 binding protein (LGALS3BP) [88], which reflects the intricacy of the IFN/ISGs signaling pathway.

### 3.2. The Induction of IFN and ISG Expression in HIV-Infected Macrophages

The main HIV-1 PAMPs comprise different viral nucleic acid molecules that are produced during the replicative cycle. Several cytoplasmic sensors, such as IFI16 and cGAS, can recognize HIV-1 DNA [52,89]. Both sensors can activate the adapter protein stimulator of interferon genes (STING) [90]. Activated STING recruits and induces TANK-binding kinase 1 (TBK), which phosphorylates IRF3, IRF7, or nuclear factor kappa-B (NF-κB), finally leading to the synthesis of type I IFN [65,91]. Although, the sensing of HIV-1 and induction of the synthesis of type I IFN can occur via recognition by TLR-7, the STING pathway has a preponderant role in infected myeloid cells, including macrophages [90,92]. Recently, McCauley et al. reported that CD4^+^ T cells, dendritic cells, and macrophages with HIV-1 provirus are activated during innate immune signaling through the type I IFN response [93]. This immune activation requires HIV-1 provirus transcription, including the expression of unspliced HIV-RNA. McCauley et al. proposed that the post-transcriptional regulation complex intrinsic to HIV-1 RNA is detected as a danger signal by the innate immune system and the proviruses do not need to be replication-competent to contribute to inflammation [93]. In this context, it was reported that macrophages can sense intron-containing HIV-1 RNA and activate other adapter proteins known as mitochondrial activator of virus signaling (MAVS) downstream of the sensors RIG-1 and MDA5 [22]. This pathway promotes a pro-inflammatory state that is dependent on type I IFN, and in co-culture of macrophages with T cells trigger the upregulation of inhibitory receptors, which leads to the exhaustion of the immune response [22]. Similarly, macrophages have been noted to trigger the expression of a broad range of ISGs just a few hours after infection, and this response does not require HIV-1 RNA reverse transcription [94]. Indeed, it was demonstrated that CD4^+^ T cells, monocytes, and macrophages infected with HIV-1 display different ISG expression profiles following type I IFN stimulation [53]. Interestingly, although CD4^+^ T cells, monocytes, and macrophages share 104 common ISGs, many of which are evolutionarily conserved in mammals and exert antiviral effects, both the number and magnitude of upregulated ISGs are higher in macrophages [53]. Combined, these data highlight that macrophages (HIV-1-target cells) can activate IFN/ISG responses through the sensing of the different HIV-1 PAMPs and further indicate that this activation does not necessarily lead to virus clearance. However, it can influence different aspects of the immune response to HIV-1, although how this occurs remains poorly defined [95]. In macrophages, this response could have unique characteristics that affect HIV-1 transmission and may potentially contribute to HIV-1 latency and reactivation.

## 4. The Relevance of the IFN/ISG Response in HIV-1-Infected Cells

Whether IFNs hinder or facilitate the progression of HIV-1 disease is controversial, while the effects of IFN-based therapy are variable [96]. The results of some in vivo studies on type I IFN production and their effect on HIV-1 infection have indicated that the IFN response is usually ineffective at suppressing viral activity, mainly due to intrinsic factors of viruses [97,98]. Indeed, in 2013, Hardy et al. showed that IFN-α was highly abundant in the peripheral blood of non-treated HIV-1-positive patients presenting chronic infection, whereas in HIV-positive patients with adherence to ART and in whom viral replication was suppressed (<50 copies/mL in plasma for >2 years), the IFN-α levels were significantly reduced [99].

For ISGs, it was proposed that their continuous expression during chronic infection can directly contribute to an increase in systemic inflammation [96]. Some researchers have additionally postulated that the progression to AIDS does not happen in natural SIV hosts owing to the downregulation of ISGs and because systemic immune activation is maintained for only a few weeks after infection [96,100]. Although controversial, an intact IFN response during acute lentiviral infection is thought to be crucial for viral control [101]. Moreover, elite controllers (HIV-positive patients who have maintained their viremia between undetectable and very low levels for 1 to 10 years without ART [70]) maintain elevated IFN-α levels in comparison with infected patients who adhere to ART [90,102]. This could be due to an innate intracellular mechanism related to IFN signaling that is mediated by ISG antiviral effectors such as TRIM5α [70,103].

A recent study revealed that plasmacytoid dendritic cells (pDC) can inhibit the establishment of HIV-1 latency in CD4^+^ T cells in vitro and in cells collected from people living with HIV who are undergoing ART through a mechanism mediated by type I IFN. However, once established, latency can be reversed by IFN-α, but not by others type I or type III IFN [104].

Relatively few studies have been undertaken investigating the different ISGs and other non-IFN cytokines using ex vivo or in vitro models. Hence, in this review, we have summarized how the different PAMPs associated with HIV-1 are sensed and their possible contribution to the inflammatory process (Table 1). The information presented offers some details of interest, including the production of powerful pro-inflammatory cytokines such as IL-6, TNF-α, and IL-1β, as well as the chemokine CXCL10, which is an ISG [64,89,105], and may help explain the immediate consequences of activating IFN/ISG signaling and its impact on the overall immune response. Two studies are highlighted. McCauley et al. observed that in response to transduction with HIV-1, macrophages upregulated the expression of the CD86 and HLA-DR receptors, an effect that was dependent on the level of HIV-1 transcription. This change could condition the future participation of macrophages in lymphocyte activation [93]. Additionally, Akiyama et al. reported that HIV-1-infection-induced monocyte-derived macrophages (MDM) activation results in T cell dysfunction by CD160 increase, which is correlated with HIV-1 disease progression and the functional impairment of T cells [22].

In addition to the above information, two key aspects of HIV-1 infection specifically highlight the importance of the IFN/ISG response:

- Type I IFN responses are thought to be the main selective pressure for the emergence of HIV-1 genotypes (transmitted/founder [T/F] variants) that initiate the infection process in humans. Different groups have reported that the T/F virus is more resistant to Type I IFN compared with the virus present during the chronic phase of infection [51,109,110]. T/F viruses can usually infect CD4^+^ T lymphocytes but not macrophages. However, the virus can eventually infect macrophages when they express an envelope with a high affinity for CD4. Nevertheless, the molecular mechanisms underlying the IFN-induced restriction of HIV-1 infection and how the virus evolves its tropism to macrophages remain unknown [39,111].
- Lentiviruses, and HIV-1 in particular, have developed accessory proteins and several strategies to counter ISG activity [70]. Viral proteins include viral infectivity factor (Vif), which inhibits the antiviral factor APOBEC3 [67]; viral protein X (Vpx), which inhibits SAMHD1 [69,112]; and viral protein unique (Vpu); which inhibits tetherin [113].

Given the above observations, the following questions remain unanswered: Is there some setting under which an optimum balance between a maximum upregulation of the viral restriction factors and a minimum upregulation of activating immune-related ISGs during HIV-1 infection can be achieved? Furthermore, could these effects influence or help in the reduction of HIV-1 reservoirs?

## 5. The Regulation of the IFN/ISG Signaling Network in HIV-1-Infected Cells

As discussed above, the fact that antiviral factors cannot restrict initial HIV-1 replication in host cells, indicates that the virus has developed successful strategies to overcome such restrictions [114]. Nevertheless, the balance between IFN signaling and ISG production is critical for post-HIV-1 infection responses. The autocrine and paracrine antiviral resistance states induced by the IFN response are fine-tuned by opposing promotive and suppressive signals [115], which leads to changes in the cellular proteome [56] and induces a rapid and effective antiviral response, while restraining the magnitude and length of the response [115].

### 5.1. Classical Mechanism

**Figure 1:**
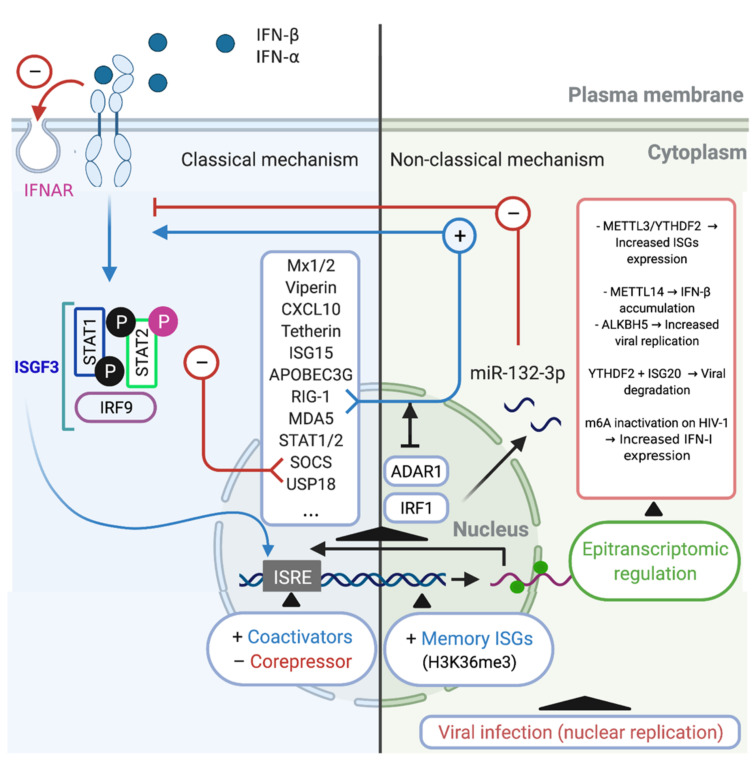
Classical and non-classical mechanisms of regulation of IFN/ISG signaling network. Classical regulatory mechanisms (**Left**). The type I IFN may conduce to activation of the transcriptional complex ISGF3, which is translocated to the nucleus where induces the expression of hundreds of ISGs such as Mx1/2, Viperin, CXCL10, Tetherin, APOBEC3G, RIG-1, MDA5, STAT1/2, SOCS, and USP18 [53]. SOCS and USP18 act inhibiting the IFN/ISG signaling pathway, in addition to the phosphorylation (Ser287) of STAT2 and the downregulation of cell surface IFNAR (red arrows (−)). Another classic mechanism occurs at the transcriptional level, it is constituted by the interaction of ISGF3 with coactivators and/or corepressors that recognize ISG response elements (ISRE), modulating the expression of ISGs. Non-classical regulatory mechanisms (**Right**). One of these non-classical mechanisms is performed by ADAR1, which is an ISG that can control IFN/ISG signaling by prevent RIG-1 and MDA5 sensing of viral RNAs (blue arrow (+)). Another non-classical mechanism is performed by miR-132-3p, which negatively regulates the IFN/ISG signaling interfering with the gene expression of IRF1 (red arrows (−)). Another example of non-classical regulation of IFN/ISG signaling is performed by an epigenetic mechanism, called “innate memory”, which favors an accelerated recruitment of RNA polymerase II and transcription/chromatin factors associated via H3K36me3, promoting mRNA transcription of associated molecules of the IFN/ISG signaling pathway. Finally, a recently described non-classical mechanism of regulation of IFN/ISG signaling pathway, involves the epitranscriptomic regulation through of the m6A-machinery, controlling the fate of IFN and ISG mRNAs for degradation or translation [115,126,127,128]. Figure created with Biorender.com (accessed on 15 July 2021).

The IFN-α/β induce the activation of the transcriptional complex IFN-stimulated gene factor 3 (ISGF3), which in turn is translocated to the nucleus where induces the expression of hundreds ISGs, such as, Mx1/2, Viperin, CXCL10, Tetherin, APOBEC3G, RIG-1, MDA5 (Table 1, [22,64,89,93,94,105,106,107,108]). Additionally, these regulatory mechanisms include the induction of negative regulators such as SOCS and USP18 [79,112,116]. However, the downregulation of the IFN-α/β receptor on the cell surface is considered to be the most specific and rapid regulatory response [117,118]. Another important mechanism involves STAT-dependent regulation. STAT2 acquires transcriptional activity upon tyrosine phosphorylation (Tyr690), whereas serine phosphorylation (Ser287) of STAT2 negatively regulates the IFN response. Meanwhile, phosphatase-dependent STAT1 dephosphorylation constitutes an important negative regulatory event that is central for titrating the IFN response [119,120]. At the transcriptional level, the transcriptional factor ISGF3, a complex that includes IRF9 and STAT1/2, binds to ISREs within the promoter regions of ISGs [121], recruiting various chromatin remodeling factors [122], transcriptional coactivators [123], and corepressors [124], which can either promote or inhibit ISG transcription [120]. In addition to these mechanisms, recent studies utilizing sequencing and proteomics technologies have defined antiviral effectors that are IFN-stimulated in a non-classical way, which have additional consequences of IFN stimulation [56,125]. These classic mechanisms are summarized in (Figure 1 Left).

### 5.2. Non-Classical Mechanisms

In macrophages, recent research has shown that during HIV-1 infection, IFN/ISG signaling can be modulated by intrinsic cellular IFN/ISG-dependent mechanisms. This process is mediated by the p150 isoform of RNA-specific adenosine deaminase (ADAR1), which is a regulator of innate immune activation and likely also of viral susceptibility in primary myeloid and lymphoid cells [129]. ADAR1 catalyzes the deamination of adenosine to inosine in viral and cellular RNA [130], which is Type I IFN inducible [131], and can also facilitate HIV-1 replication in primary CD4^+^ T cells [132]. The mechanism through which the absence of ADAR1 blocks viral replication and HIV-1 protein synthesis in myeloid and lymphoid cells was recently elucidated. For macrophages, Pujantell et al. found that ADAR1 modulates the recognition of foreign RNA in the cytoplasm, such that when ADAR1 is silenced, MDA5 and RIG-1 recognize HIV-1 RNAs, leading to the activation of MAVS and TBK as well as their downstream effectors IRF3 and IRF7 (these can also be induced by Type I IFN and ISGs), thereby inhibiting HIV-1 infection [129]. This mechanism likely explains how macrophages become susceptible to HIV-1 infection (Figure 1 Right).

A non-classical Type I IFN-stimulated pathway and in the context of epigenetic regulation includes the use of alternative transcription start sites, which induces alternative splicing, leading to unstable transcripts or those with different efficiency during translation [56]. Similarly, Type I IFN stimulation can reportedly disrupt the expression of microRNAs [133] or long noncoding RNAs. The latter, through directly binding to chromatin remodeling complexes, can influence gene expression [134] or serve as a scaffold for the formation of ribonucleoprotein complexes with antiviral activity [56] (Figure 1; Right). For instance, during H1N1 influenza A virus (IAV) infection, the upregulation of miR-132-3p expression promotes IAV replication by inhibiting IAV-induced IFN-α and IFN-β production and ISG expression through its target gene *IRF1* [135]. Although, against HIV-1 infection, several cellular miRNAs were described with the ability to inhibit HIV-1 infection/replication in macrophages, stands out the miRNAs that targeting the viral genome (e.g., miR-28, miR-125b, miR-150, miR-223, and miR-382) [136,137] and host cell proteins required for viral replication (e.g., miR-155 and miR-146a) [137,138]. Although, it has recently been described that in primary human macrophages, miR-155 is induced by agonists of TLR3 and TLR4, which is associated with HIV inhibition, and that also miR-155 could target the 3′-UTR of TRIM32 mRNA and facilitate HIV latency by repressing activation of NF-κB [137,139,140]. A putative direct relationship between miRNAs with a HIV-1-restriction activity and IFN were recently suggested, where inhibition of type I and type III IFN by HIV-1 infection seems to be responsible for modulating the expression of these miRNAs, and on the contrary, treatments of type I IFN in primary human monocytes and macrophages induced the expression of the HIV-1-restriction miRNAs, stands out the miR-28, miR-125b, miR-150, and miR-382 [141].

In the same epigenetic regulation context, recent evidence indicates that the innate immune system also has some capacity for memory or so-called trained immunity [142]. For instance, a recent genome-wide study revealed that IFN stimulation confers transcriptional memory that permits faster and greater ISG transcription in mouse embryonic fibroblasts (MEFs) and bone marrow (BM)-derived macrophages [143]. This memory was not due to enhanced IFN signaling or retention of transcription factors on ISG promoters but was instead attributed to the accelerated recruitment of RNA polymerase II and transcription/chromatin factors associated with the acquisition of the histone H3.3 and H3K36me3 chromatin markers on memory ISGs. This mechanism represents a readjustment of gene expression programs in the cell to accommodate changing environments [143].

### 5.3. Alternative Mechanism: Epitranscriptomic Regulation

Some evasion mechanisms developed by HIV-1 through co-evolution could directly modulate the expression of IFN/ISG signaling network-related genes at the transcriptional level. Recently, it was reported that the replication of RNA viruses can be controlled through epitranscriptomic mechanisms [144]. In the context of HIV-1 infection, some ISGs are known to undergo DNA methylation in early and chronic stages of infection [145], while direct RNA-editing events also play important regulatory roles at the post-transcriptional level, including in the immune system [142]. In the latter case, the loss of the enzyme ADAR1 was shown to lead to spontaneous, MDA5-dependent interferon production and impaired editing of endogenous long double-stranded RNAs [146]. However, epitranscriptomic-based RNA modifications, which until very recently were considered to be static and unalterable after their covalent attachment, could have an important role in many cellular processes, such as DNA and histone modifications [147]. Coding and noncoding RNAs can undergo more than 100 distinct chemical modifications, some of which (e.g., N6-methyladenosine [m6A], 5-methylcytidine [m5C], inosine [I], pseudouridine [Ψ], N1-methyladenosine [m1A], and 5-hydroxylmethylcytidine [hm5C]) occur internally in eukaryotic mRNAs and can influence their metabolism and function [142,148]. m6A methylation is the most frequently observed of these internal mRNA modifications in eukaryotes [148], and can influence mRNA splicing, translation, and stability [149]. Recent evidence also indicates that it may constitute a novel hallmark in virus-host interactions [149,150,151].

The m6A modification on mRNA is post-transcriptionally added, disengaged, and recognized by methyltransferases (writers), demethylases (erasers), and m6A-specific binding proteins (readers), respectively [152]. This modification allows for rapid gene expression responses and control of protein production [153]. The m6A methylation complex in mammals includes methyltransferase-like proteins (METTL) 3 and 14, Wilms tumor 1-associated protein (WTAP), and KIAA1429 [154,155]. The removal of m6A is facilitated by fat mass and obesity-associated protein (FTO) [156] and alkB homolog 5 (ALKBH5) [157]. Writers and erasers determine the prevalence and distribution of m6A on the mRNA, while the readers mediate m6A-dependent functions. Members of the family of YT521-B domain (YTHDF1-3 and YTHDC1-2)-containing proteins are direct m6A readers and contain a conserved m6A-binding pocket [149,158,159].

Viruses can induce changes in the distribution of m6A modification on cellular mRNAs [160]. In the context of the IFN/ISG signaling network and nucleus-replicating viruses, Winkler et al., showed that a fast turnover of cellular mRNA characteristics was mediated by m6A modification and that this could critically affect responses to external stimuli. The authors further observed that in infected fibroblasts, the presence of m6A modifications on IFN-β mRNA led to a fast turnover of this mRNA, which negatively affected the Type I IFN-mediated response and facilitated viral propagation [115]. A different study showed that IFN-β1 production triggered by human cytomegalovirus (HCMV) is controlled by METTL14 and ALKBH5 [126] (Figure 1 Right).

Meanwhile, a relationship between ISGs and hepatitis B Virus (HBV) has also been reported. ISG20, a 3′-5′ exonuclease, was reported to selectively recognize m6A-modified HBV transcripts and process them for degradation. This effect was critically regulated by the m6A reader protein YTHDF2 and methylation at nucleotide A1907 (a unique m6A site within HBV transcripts), showing that this process is a critical regulator of the IFN-α mediated decay of HBV RNA [127].

Regarding m6A modification and pro-inflammatory (M1) and anti-inflammatory (M2) macrophage polarization, it was reported that YTHDF2 can degrade m6A-modified STAT1 mRNA, thereby regulating glycolysis and M1 macrophage polarization. The underlying mechanism is dependent on the direct interaction between YTHDF2 and RNA-binding motif 4 (RBM4), the latter of which is known to modulate the proliferation and expression of inflammatory factors [161]. In contrast, it was noted that METTL3 is specifically upregulated following the M1 polarization of mouse macrophages. METTL3 directly methylates STAT1 mRNA, thereby increasing its stability and subsequently upregulating STAT1 expression [162]. These data suggest that epitranscriptomic (m6A)-mediated regulation could be an important mechanism during viral infection and the IFN/ISG response and is also related to the IFN/ISG response in the differentiation of macrophages (Figure 1 Right).

Considering that in HIV-1 infection, HIV-1 mRNA is known to contain multiple m6A modifications [163], and that these m6A modifications influence not only the translation of HIV-1 genes (RNA to protein) but also HIV-1 cDNA synthesis (RNA to DNA). Additionally, m6A reader proteins (YTHDF1-3) can both positively and negatively affect different steps in the life cycle of the virus [5,164,165,166]. A recent study demonstrated that in myeloid cells (monocytes and macrophages) the m6A modification in HIV-1 RNA can suppress Type I IFN expression, and when the m6A modification is altered/defective, the affected RNA is sensed by RIG-1 [128]. However, to date, no studies have directly linked the IFN/ISG response and the m6A modification in macrophages that serve as replication-competent latent HIV-1 reservoirs.

## 6. Conclusions and Future Perspectives

Macrophages present a specific intracellular innate immune response that comprises the induction of antiviral cytokines, including type I IFN (IFN-α/β), which culminates in the expression of ISGs covering a wide range of biological activities.

However, the IFN/ISG response against HIV-1 infection has only been partially defined and remains incompletely understood. The flexibility already described for the combination of pleiotropic and specific interactions within the antiviral defense system associated with the IFN/ISG signaling network [85] may explain the scenarios possible during HIV-1 infection. This review has focused on the relationship between the IFN/ISG signaling network and the susceptibility of target macrophages, and their contribution to the formation of replication-competent HIV-1 reservoirs in infected macrophages. The proposed mechanism considers the regulation process of IFN/ISG signaling network through an epitranscriptomic regulation.

Given these facts, the following questions remain outstanding: Can HIV-1 infection in macrophages induce an imbalance in the IFN/ISG signaling network? Could this imbalance determine whether an active HIV-1-infected macrophage becomes a replication-competent latent HIV-1 reservoir? We propose that virus–host interactions alter the epitranscriptomic regulation of the IFN/ISG signaling network in macrophages to promote an imbalance in this network as well as in viral replication during the initial infection. With time, this imbalance may drive a replication-competent latent HIV-1 infection.

**Figure 2:**
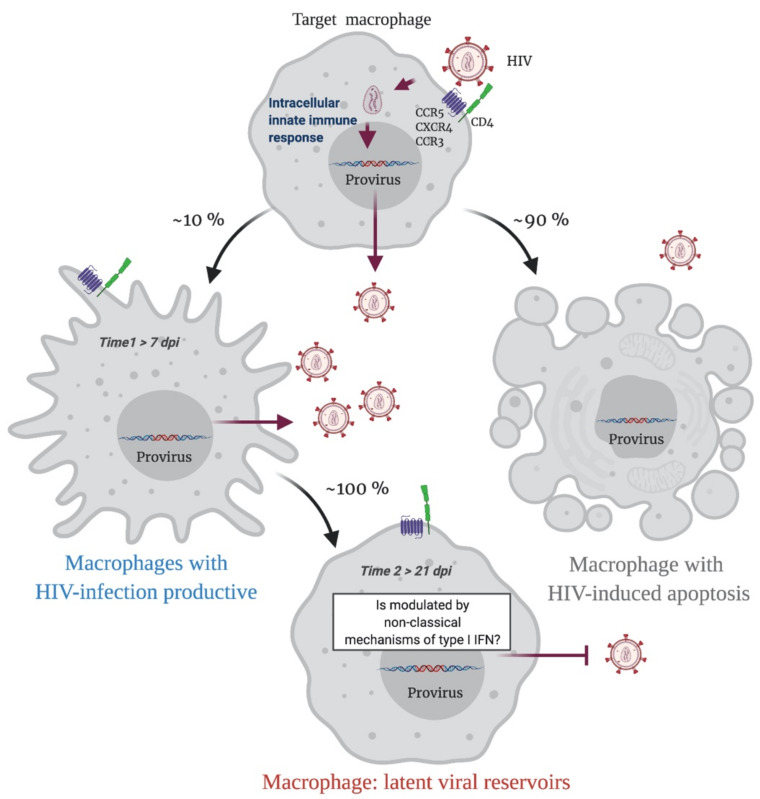
A Representative scheme of putative sceneries during the HIV-1 infection in macrophages. Initially, an HIV-1 target macrophage will be infected, a replicative cycle will be completed, and the host genome will have an integrated HIV-1 proviral DNA. This triggers an innate intracellular immune response, and the infected macrophage has two possible destinations. Apoptosis will result in 90% of HIV-infected macrophages, while 10% of cells will survive and continuously produce viral particles. This last phenomenon is probably determined by a modulation in the IFN/ISG signaling network that fails to restrict viral replication (**Time 1**). Over time, this modulation will possibly be sustained by non-classical mechanisms of type I IFN regulation such as epitranscriptomic regulation, which will allow the cell to become a replication-competent latent viral reservoir (**Time 2**). Figure created with Biorender.com (accessed on 15 July 2021).

In summary, when a HIV-1 proviral DNA is integrated into the macrophage genome, an immune response is triggered, and infected macrophages have two possible destinations. Apoptosis will result in 90% of HIV-infected macrophages, while 10% of cells will survive and continuously produce the virus. This last phenomenon is probably determined by a modulation in the IFN/ISG signaling network, that fails to restrict viral replication (Time 1 > 7 dpi; Figure 2). Over time, this modulation will possibly be sustained by non-classical mechanisms of type I IFN regulation such as epitranscriptomic regulation, which will allow the cell to become a replication-competent latent viral reservoir (Time 2 > 21 dpi; Figure 2).

## Figures and Tables

**Table 1:** Induction of IFN/ISG and pro-inflammatory molecules, reported downstream of HIV-1 sensing in macrophages.

Cell Type	IFNs	ISGs	Other Cytokines and Costimulatory or Activation Molecules	Reference
THP1, Primary human MDMs	IFN-β	TREX1, CXCL10	CXCL10	[105]
Primary human MDMs THP1/MDM Murine macrophages	IFN-β IFN-λ1	CXCL10, IFI16	IL-6, TNF-α, IL-1β	[89]
THP1, Primary human MDMs	IFN-β IFN-α2 IFN-α4	ISG56, ISG15, APOBEC3G	CXCL10, IL-6, IL-12α	[64]
Primary human MDMs	IFN-α IFN-β	No reported	No reported	[106]
(HIV-1)-infected macrophages from patients with HAND	IFN-α2 IFN-α1 IFN-β	RIG-1, MDA5	No reported	[107]
Primary human MDMs	IFN-β1	ISG15	CD86, HLA-DR	[93]
Primary human MDMs	IFN-β IFN-α2	CD169/Siglec1, CXCL10	CD160, CXCL10, MCP-1, IL-15, VEGF	[22]
Primary human MDMs	IFN-λs	Mx2, Tetherin	No reported	[108]
Primary human MDMs	IFN-β	>17 ISGs upregulated, i.e., Mx1, Viperin, ISG15, CXCL10, TNFSF10 (or TRAIL)	CXCL10	[94]

Abbreviations: monocyte-derived macrophages (MDMs), human macrophage-like cell line [phorbol12-myristate13-acetate (PMA)-differentiated THP1 cells] (THP1/MDM), Tumor necrosis factor (ligand) superfamily, member 10 (TNFSF10), TNF-related apoptosis-inducing ligand (TRAIL).
